# Voices of those living with type 2 diabetes in Belize: barriers to care before and during the COVID-19 pandemic

**DOI:** 10.1186/s12939-023-01987-3

**Published:** 2023-08-24

**Authors:** Lindsay P. Allen, Lucia Ellis, Christophe Engleton, Valerie Lynette Valerio, Andrew R. Hatala

**Affiliations:** 1 Department of Community Health Sciences, Max Rady College of Medicine, University of Manitoba, 221 Human Ecology Bldg, Winnipeg, MB R3T 2N2 Canada; 2 Belize Diabetes Association, Belize City, Belize

**Keywords:** Type 2 diabetes, Barriers to care, Belize, Central and South America, COVID-19

## Abstract

Belize has the highest national prevalence of type 2 diabetes (T2D) of Central and South America, and fifth direst in the world. T2D is the leading cause of death in Belize, a country facing burdens of increasing prevalence with few resources. Since March of 2020, the COVID-19 pandemic has exacerbated the difficulties of those living with T2D in Belize. To address T2D issues in Belize, our interdisciplinary research team explored the barriers to care and self-management for adult patients with T2D in Belize prior to and during the COVID-19 pandemic.

Research relationships between Canadian (ARH) and Belizean (LE) authors have been ongoing since 2016. Together we used a qualitative Constructivist Grounded Theory design generating knowledge through 35 semi-structured patient interviews, 25 key informant discussions, and participant observation with field notes between February 2020 to September 2021. We used Dedoose analysis software for a systematized thematic coding process, as well as iterative verification activities. Findings revealed several barriers to care and self-management, including: 1) the tiered health and social care system with major gaps in coverage; 2) the unfulfilled demand for accurate health information and innovative dissemination methods; and 3) the compounding of loss of community supports, physical exercise, and health services due to COVID-19 restrictions. In the post-pandemic period, it is necessary to invest in physical, nutritional, economic, and psychosocial health through organized activities adaptable to changeable public health restrictions. Recommendations for activities include sending patients informational and motivational text messages, providing recipes with accessibly sourced T2D foods, televising educational workshops, making online tools more accessible, and mobilising community and peer support networks.

## What is known about this topic

- Social, familial, and peer supports are key to sustaining type 2 diabetes self-management, but these were confined during the COVID-19 pandemic.
- Regular glucose monitoring, physical exams, and lifestyle behavior modification are part of the standard of care for patients, but these became even less accessible during the COVID-19 pandemic.
- Without adequate screening, treatment, and self-management education and support, patients are at increased risk for co-morbidities and mortality; thus, the global COVID-19 pandemic impacted people living with type 2 diabetes not only due to risks involved with contact with the virus, but also the risks involved with restrictions.

## What this paper adds

- Verbatim expressions of urgency from people living with type 2 diabetes in Belize during the COVID-19 pandemic.
- Complexity and nuance for policy decision making around addressing chronic illness under pandemic and everyday contexts.
- Community-based innovative recommendations for practice and implication suggestions.

## The severity of type 2 diabetes in Belize

Epidemiologists have warned that diabetes has surpassed the status of ‘epidemic’ and that “the emergence of type 2 diabetes as a global pandemic is one of the major challenges to human health in the twenty-first century” [44], p.1432). In Belize, a country considered among the least developed in Central America, type 2 diabetes (T2D) is the foremost cause of death [43, 46]. The prevalence is now over 17% in the adult population, having increased from 10% since 2010 [46]. The national prevalence is highest of all countries in Central and South America, and fifth direst in the world [46]. Little is known about how T2D populations in Belize are managing their day-to-day care, and especially how management was impacted by the COVID-19 pandemic.

Twenty-five percent of Belizeans are still waiting for national health insurance, which began a spotty roll-out in 2003 [6, 29]. A 2018 random sample found 33% of Belizeans to be in the prediabetes range, with 8.5% previously undiagnosed [20]. Hospitalization rates for associated complications (i.e., cardiac arrest, cerebral vascular accident, renal failures, lower limb amputations, vision damage, vision loss) are also increasing in Belize [29, 43].

A 2017 mixed-methods study in the villages of southern Belize found that 79% of people were living far below the poverty line, and T2D was poorly controlled in 74% of diagnosed patients [12]. A 2014 diabetes screening via a major employer in Belize found 50% of employees to have elevated glycemic levels [15]. Throughout Belize, people with diabetes often go without adequate proteins, complex carbohydrates, glucometers, testing strips, and oral medications [7, 12, 15].

The emergence of COVID-19 has had severe implications for those living with T2D [39]. People are more likely to experience acute symptoms and complications from the virus and have disproportionately higher COVID-19 fatalities, if they have T2D [39]. This is an urgent matter of health equity, considering that T2D is most prevalent in structurally disadvantaged racialized groups [4, 11, 14, 26].

There are some clinical studies of T2D and COVID-19 co-morbidity, but there are very few qualitative studies globally on T2D patients’ experiences during the pandemic. There has been one study in Ethiopia [25] and one in Denmark [21, 22] which document negative psychosocial and economic impacts of the pandemic on T2D self-management. There are major knowledge gaps about T2D self-care and management in Belize, as well as diverse treatment options and support [1]. There are also gaps where advancements in theory are needed to understand how this pandemic has disproportionately impacted people living with pre-existing chronicity. This study builds understanding of the barriers to care and self-management for adult patients with T2D in Belize prior to and during the COVID-19 pandemic, in hopes to inform practice, policy, and planning locally and globally.

## Methods

### Research network and team

This research was initiated by the Belize Diabetes Association (BDA) in order to better understand the lived experience and care needs of people living with T2D in Belize, as a first step in developing appropriate interventions. The BDA is a non-governmental organization that provides educational supports and subsidized medical supplies to Belizeans living with diabetes. The World Diabetes Foundation provided funding for the research, while the Belize Ministry of Health (MoH) and the Pan American Health Organization (PAHO) acted as collaborators. A Steering Committee involved 14 people from the BDA, the MoH, PAHO, local health professionals, and a project coordinator. The research team consisted of two researchers from the University of Manitoba – who provided training in qualitative research – and three local interviewers. Research relationships between Belizean and Canadian team members have been ongoing since 2016.

### Design

Our interdisciplinary research team operated from a pragmatist paradigm and undertook a qualitative design to explore and understand people’s lived experiences. Specifically, we followed Constructivist Grounded Theory (CGT) methodology since it positions participants as experts of their own lives with valuable, intimate, first-hand knowledge of the topic area [8–10, 17, 18]. Charmaz described CGT as applying a critical lens to examine how power works in and through societally constructed institutions, systems, and processes, to relate that understanding to social justice issues (e.g., barriers to care, poverty-induced diseases, syndemics of chronicity), and to illuminate the imbalanced distribution of suffering across study populations [8]. This methodology allows for local people to directly inform theories and understandings about their lives, with knowledge being constructed from their accumulated lived experiences [8].

### Sample

Purposive and theoretical sampling [17, 18] were used to recruit adults participants 18 years or older with diagnoses of T2D from the five most populated districts of Belize (Toledo, Stann Creek, Cayo, Belize City, and Corozal). The purposive sampling process began with the leadership of the local Steering Committee and research coordinator who mobilized nationwide cooperation through their networks of relevant stakeholders. Theoretical sampling carried on throughout the pandemic as we narrowed in on emerging themes and theory. The final sample demonstrates the diversity of the country with participants of Creole, Garifuna, Mayan, Mestizo, and East Indian ethnicities. Conversations were also held with 25 key informants, including personnel at National Institute of Culture and Heritage, Ministry of Health, National Health Insurance, Belize Diabetes Association, Punta Gorda Polyclinic, Independence Health Authority, San Antonio Clinic, and University of Belize. Key informants included physicians, nurses, community health workers, health educators, health directors, administrators, and the Steering Committee. Thirty-five adult Belizeans (27 women, 8 men) with T2D residing in Belize were enrolled in this study, and no one declined or dropped out. The mean age of the participants was 54 years old, with an age range of 34 to 89. Table 1 presents the demographic characteristics of the study participants.

**Table 1:** Demographic characteristics of the study participants

**Age range in years old**	*n* (%)
30–39	3 (9)
40–49	6 (17)
50–59	17 (49)
60–69	6 (17)
70–89	1 (3)
Missing observations	2 (6)
**Sex**	***n* (%)**
Female participants	27 (77)
Male participants	8 (23)
**Ethnic background**	***n* (%)**
Creole	13 (37)
Mayan	4 (11)
Garifuna	6 (17)
Mestizo	6 (17)
East Indian	5 (14)
Other	1 (3)
**Occupation**	***n* (%)**
Employed outside home	7 (20)
Worked inside home	18 (51)
Retired	10 (29)

### Data generation

The primary data generation method was semi-structured audio-recorded interviews lasting thirty to ninety minutes. Thirty-five interviews were conducted between February 2020 and September 2021, mostly face-to-face, though there was one phone interview during the strictest pandemic measures of isolation. Interviews took place in clinics, community centres, and homes, to the preferences of participants. Most participants were comfortable being interviewed in English (the national language). Two were interviewed with local translators (one in Mayan and one in Spanish). As the team consisted of male and female, Canadian and Belizean researchers between twenty and sixty years of age, interviews were conducted according to what the team felt would make participants most comfortable. The interview guide was based on the Diabetes Quality of Life Questionnaire which was pretested in February and March of 2020 for cultural saliency and modified to include feedback from the Steering Committee and the first eleven interviewees. Secondary methods included site visits, discussions with key informants (in person and online), and participant observation with field notes [8, 9, 17, 18]. All research was approved by the University of Manitoba Human Research Ethics Board (HS23313 (H2019:406)) and (HS23931(H2020:229)). The local Steering Committee and stakeholder partnerships (i.e., Belize Diabetes Association) ensured all ethical and cultural protocols were followed and appropriate to the context of Belize.

### Data analysis

In accordance with the Constructivist Grounded Theory framework, we iteratively developed themes through first highlighting literal codes and open codes, which progressively became more focused codes with sub-codes in systematized order (i.e., phrase-by-phrase and line-by-line) using Dedoose qualitative analysis software [9, 37]. This involved scrutinizing data for emerging patterns, organizing them into main themes and subthemes, analyzing interconnected themes and comparable themes, and attending to underlying processes, assumptions, and meanings [9, 37]. The first author (LPA) was the primary coder, the fifth author (ARH) verified the coding, and all authors engaged in iterative discussions of emerging themes, as well as reviewed and verified a series of drafts of the article [9, 31, 37]. Various drafts were reviewed by the research team and the Steering Committee. Participants were asked whether they preferred to use pseudonyms for anonymity or keep their real names attached to their contributions for acknowledgement; pseudonyms were assigned to all participants as per their wishes [31].

The rigor and trustworthiness of the analysis was ensured through: 1) transcribing interviews verbatim, integrating interviewer notes that attended to body language and emotional details; 2) continuing to collect data to and beyond the point of data saturation; 3) discussing and reviewing coding and analysis with and among Belizean Steering Committee and research team members in an iterative process; 4) practicing reflexivity and introspective integrity on a continuous basis; 5) considering how each step in the decision trail might impact participants before making decisions; 6) scrutinizing if the emerging theory makes sense from within its context; and 7) looking for any data that might disprove, change, or add to the emerging theory [37]. Though we had reached a point of data saturation after approximately twenty interviews, we continued doing interviews with more diverse participants to ensure no new themes emerged [28, 41].

## Results

Interviewees expressed that they could not reliably access treatment and self-management supports before COVID-19, and this was exacerbated during the pandemic. They often had difficulty getting accurate information about T2D, let alone solutions applicable to their socio-economic realities. Three main themes emerged from interviews and key informants: 1) the health care system is tiered and has major gaps in coverage; 2) the unfulfilled demand for accurate health information and innovative dissemination methods; and 3) the compounding effects of loss of health services, community supports, fitness, and nutrition due to COVID-19 restrictions.

### "People who can afford it": A tiered health care system and the cost of poverty

Many places do not have public clinics, and where they exist, there are long waiting lists to get into the public system. Natalia, a working mother of three in Belize City, was concerned for those without basic coverage under the National Health Insurance (NHI): “The Ministry should have more NHI that people can go because a lot of people want NHI, and they cannot. It’s hard ‘cause not everyone has the finance to [get care otherwise].” While some participants were relieved just to be included in NHI, others were willing to pay for private health services despite having very limited financial resources. Josefina, an elderly woman living on very little income, strongly preferred the private clinic. She explained, “Because sometimes I go [to the public clinic], and it’s a lot of patients to wait for. Sometimes they don’t have the medication.” Yvonne similarly stated, “People who can afford [to go to the private clinics] … You get better medication than the public clinics, and you don’t have to wait that long time.” Participants described experiences of being ill with standing room only in the waiting room. Others described travelling a long way to a clinic only to find no doctors on duty.

To save money and to adjust for COVID-19 related shortages, several people reported taking their medication once a week instead of once a day. Only three participants said that they were testing their blood glucose levels regularly. Despite daily blood glucose testing being considered a part of routine T2D self-management, this omission was due to the cost and availability of glucometers and test strips. Maurice, for example, worried about this: “With my sugar going up and down, I can only wonder how other people feel, who do not have a glucometer, and have these strange feelings in their body.” Participants reported episodes of trembling, vertigo, nausea, headaches, weakness, numbness, confusion, fatigue, helplessness, feelings of imminent doom, fear of death, and other fears associated with T2D and uncontrolled glucose levels.

Some participants had adequate financial resources, and they could succeed in T2D self-management. For those with less, “self-managing” meant being alone. Felicia expressed: “We no rich, so we have to take care of ourselves. If you have to go on dialysis, you don’t have money for that! If you know a minister or know somebody like that, they’ll say ‘leave it’, and you don’t need to pay money.” She continued, “If you don’t know nobody, you have to find the money. You family going to be made to pay. Look how much it costs for dialysis! You spend all your money, and you’re still dead.” Given that T2D is known to disproportionately impact the poorest segments of society (e.g., [36, 45], addressing barriers to services and economic marginalization must be addressed simultaneously.

### "I wish they would educate people now": The need for nationwide education

When asked what they want, participants consistently said prevention and education programs; for example, people wanted to be able to access more information on nutrition and nutritious food production. Veronica pointedly asked, "They say we eat too much rice, but then what else can we eat?” Rosa forwarded her vision of a special Belizean diabetes cookbook, showcasing local foods for this purpose.

George stressed the need for lifestyle counselling, educational workshops, and peer mentorship to help people improve their conditions, rather than a single-pronged strategy of increasing dosages and dependence on expensive imported pharmaceuticals. Henry wanted diabetes workshops and fitness programs, saying, “That would be good if we had more support groups and different programs toward this diabetic thing, no? So, we could learn more about it.” His first and only source of T2D information was the doctor who diagnosed him. He felt that had he grown up with consistent educational messaging from home, school, community, and media, he felt he may never have gotten to the point he did with the disease. Similarly, Yvonne wanted Belizeans to understand more about nutrition, so diabetes could be prevented in the first place: “It is never too early to start to eat healthy. Don’t wait until you’re diagnosed with diabetes!” Yvonne believed that if given accurate information, people could better harness their own resolve to avoid T2D. This lack of information was any injury to peoples’ agency as well as their health.

As a retired nurse, Cynthia agreed: “[What] I would like to see happen is constant education because it’s limited.” When asked what worked best, she described: “Talking about their condition, talking about what to eat, the importance of taking their medication, the importance of going for their blood test in order for them to be controlled, monitored correctly.” Cynthia envisioned a program with individual counselling and community workshops with tools for people to take home, such as glucose testing supplies, monitoring logbooks, recipes, and meal planning supplies. Beyond informing people on T2D nutrition, she wanted family-inclusive cooking classes to mobilise social supports while supplying some healthy meals.

Participants called for a comprehensive program to raise societal awareness about T2D across Belize. They highlighted education for those already living with diabetes, those with prediabetes, rural and remote populations, school children, and men who were perceived as less likely to get tested. As Natalia stated, “I would want more people to check, especially the men. They’re in denial and ashamed. In Belize, the first thing come from people, oh, you have AIDS! They think hard, and they know they are diabetic, but [they don’t get tested].” Considering that social stigma around AIDS is severe enough to impact testing and help-seeking behaviour, and that T2D is commonly misunderstood, the lack of education likely has significant unknown consequences. Indeed, many commented how there are unfounded theories circulating about transmission of AIDS, COVID-19, and diabetes in Belize.

Felicia who had been diagnosed with T2D in her twenties, was concerned for young people who could still prevent the disease: “It hurt me bad. I could have done better back then. Nobody educate you, so I wish they would educate people now.” She also expressed concern for older people and people with disabilities who were often overlooked by health programs. 

Educational programming is needed to tackle the overall lack of knowledge of T2D causation, management, and strategies to prevent its onset and complications. Accounting for financial and structural constraints, which were strained further by the COVID-19 pandemic, is needed in both program intervention planning and long-term planning.

### “Because we stay locked up”: Implications of COVID-19 restrictions on T2D management

Numerous aspects of self-management and regular clinical care for people with T2D have been impacted negatively by the COVID-19 pandemic. Clinics limited their hours of operation, and routine check-ups were reduced in frequency, changing from once per month to once per six months, or even, not at all. Victoria stated, “Since this curfew happened, I was not seeing any doctor because I was not going anywhere. It's been like over a year going for two years like that.” Irma similarly explained, “Since COVID we were told to stay home, so I have not seen a doctor since last year." Participants reported that since COVID-19, they have not seen physicians, dentists, optometrists, dieticians, or foot care specialists, avoiding clinics and hospitals. A lot of people typically rely on health care provision and/or medications across the borders in Mexico, Guatemala, and the United States, who provide both regular and specialist services not available in Belize. These became no longer accessible due to border closures.

An important aspect of T2D self-management is that of establishing and maintaining physical exercise. Gyms in Belize City, however, have been closed during the pandemic, and most people cannot afford home gyms. Paula shared her difficulty in maintaining an exercise regime in isolation, “I used to have a friend [to go to the gym with], but she does not want to go anymore [because of COVID]. I used to walk with my neighbor, but now with COVID they say you are not supposed to walk beside each other.” David also said, “I was a football player and after I stopped playing football, I decided to do coaching. Since coronavirus I cannot do much training.” Veronica tried to exercise but was limited, “Just walks in the yard because the road has too much traffic.” The loss of access to social contacts negatively affected participants’ progress with their fitness goals through decreased motivation otherwise found in exercising together and being held accountable by others. Diana spoke of self-motivation as a matter of survival. She expressed, “We need to be disciplined on our own to survive COVID.” Vilma described the social poverty: “We barely talk to people, because we stay locked up because of COVID, so we don't have a lot of communication with a lot of people. My daughters don't come around us because of COVID.” People spoke about the loss of social and spiritual support because of the closure of churches.

Proper nutrition is another critical aspect self-management. Due to transportation restrictions, port closures, and price increases, participants had difficulty buying fruits and vegetables during COVID-19. Yolanda reported being “afraid a lot during quarantine” and “at home cooking a lot,” thus gaining extra weight which impeded her T2D self-management goals. Irma was one of the participants who had lost self-employment income “due to COVID the business went slow,” and many expressed the rising cost of food was worrisome. Diana explained the impacts on her income thus diet, “First it was manageable because I was working as a babysitter. My kids work too, but because of the COVID virus there is a lot of things that you cannot do. I used to be able to get fruits and vegetables, but things get harder, sometimes I don’t babysit as much.” Paula relayed her frustration, “You are supposed to buy healthy food as diabetic, and—especially when you are not making a lot of money- it is expensive. If I cook for me and my family, I would only be able to cook for one day of the week to stay healthy. I try different things but it's hard.” Hector similarly stated, “There is no fruits and vegetables, what they recommend, and I find it very expensive to manage and keep those. I do try, but I can't do it all the time.”

Many participants underscored the added mental stress and psychological pressure they felt in dealing with T2D under COVID-19 constraints. Hector recalled, “Sometimes I sit and cry, and my wife will try to get me out of that.” Vilma’s mental health was impacted by enduring the compounded isolation of having T2D during COVID, “Sometimes I feel so down, I don't even want to talk to people. But I am learning that when I feel like that, my sugar is high or low.” This again highlights the need for access to testing, medications, and nutrition. Kiran said, “We are used to working for our money, now we don't have any jobs. Since March 20th [2020] we have been living on savings. Now we are on our last… Once in a while I feel sorry for myself and wonder how I will make it… I feel I suffer from depression, because I feel bad and sad for no reason, … but not bad as to commit suicide.” Clearly there have been monumental impacts on access to clinical care, medications, fitness opportunities, nutritious foods, and social supports, as well as on mental health, due to the globalized sequelae of the COVID-19 pandemic. Overall, wellness declined throughout the duration of the pandemic, with participants’ responses increasing in severity and urgency.

## Discussion and implications

To the best of our knowledge, this is the first qualitative study with people living with T2D in Belize. Previous literature has described Belize as facing several limitations in the provision of clinical services; for example, the NHI primary care responsible for diabetes screening and management does not yet cover four of the countries’ six districts, leaving people living in Cayo, Orange Walk, and most of Corozal and Belize City districts without publicly covered health services [29]. Further, the demand for laboratory services, human resources, infrastructure, pharmaceutical supplies, and equipment has increased significantly over the past two decades, yet the health care system has failed to keep pace with this growing need [29]. Our findings add that this pre-existing strain has seriously exacerbated by the COVID-19 pandemic, which has left many people with diabetes without regular access to care due to reduced clinical capacity, lock-down measures, and medication shortages. In the past, health care efforts have been supplemented by international volunteer projects, by relatives who live abroad (typically the United States), and by the Belize Diabetes Association providing medical supplies (e.g., glucometers, testing strips) to patients at reduced costs in certain locations. Much of this typical flow of resources have been obstructed due to COVID-19 travel and port restrictions and closures. In raising awareness of these issues, we hope to alert the international community to the need for renewed efforts in the post-pandemic period.

We found that people were differently equipped to access treatment depending largely on their economic status before and during the pandemic. Participants described the health care landscape in Belize as a tiered composition of private, semi-private, and public NHI clinics. Those individuals with the resources to do so often chose to pay directly for such things as private clinics, brand name prescriptions, home care, dentistry, out-of-country surgery, gym memberships, herbal doctors, and other wellness supports [1]. Those who could not afford these items suffered more long-term, drawn-out complications. This can be linked to similar findings from a 2019 study in Peru that demonstrated socio-economic and structural factors act as barriers to individual’s capacity for T2D self-management and living well with the disease [33]. This suggests serious need to complete the NHI roll-out that was originally intended to reach all Belizeans, and to refocus on micro-economic interventions (e.g., support for small food vendors, child-care providers, entrepreneurs) for improved health equity.

The public NHI clinics were much reduced in capacity during the COVID-19 response, leaving people with T2D with less care during a more vulnerable time. People went up to two years without any kind of clinical care. While it was critical for people with uncontrolled diabetes to reduce longer times in waiting rooms given increased susceptibility to COVID-19 complications, this blanket approach created too much of a void of care, thus a more nuanced approach is needed going forward. This could include such things as in-home appointments, use of telehealth and other online supports, and exceptions to border and port closures for the purposes of distribution of medical supplies.

Belize’s pre-existing shortages of medical supplies and pharmaceuticals intensified during the pandemic. A 2021 study on barriers to diabetes self-care practices in Eastern Ethiopia showed similar findings regarding food and medicine shortages due to systemic COVID-19 changes [25]. Similar to findings from a 2021 study on older adult Haitians settled in the United States, people in Belize have been experiencing severe financial hardship that forces them to choose between buying food or buying medications [27]. These studies speak to the unintended negative consequences that COVID-19 restrictions have had on structurally disadvantaged people living with T2D.

The pandemic response required people to physically distance from others, and at times, to lock down and isolate themselves in their homes. Numerous studies conducted prior to COVID-19 have demonstrated strong positive relationships between T2D self-management behaviours and social supports [5, 13, 16, 19, 23, 25, 27, 30, 32, 38, 42]. Research conducted in Algeria, Australia, Canada, China, Denmark, Ethiopia, France, Germany, India, Italy, Japan, Mexico, the Netherlands, Peru, Poland, Russian Federation, Spain, Turkey, the UK and the United States, for example, concur that more frequent social contact has positive impact on T2D self-management behaviours, including: improved eating habits, higher frequency of physical exercise, higher frequency of foot self-examinations, higher adherence to medication schedules, reduced smoking, improved activation and motivation, and reduced emotional distress and mental problems [5, 13, 16, 19, 23, 25, 27, 30, 32, 38, 42]. Our study uniquely documents that conversely, and in the Belizean context, social isolation as experienced during the COVID-19 pandemic, was detrimental to T2D self-management practices, constricting access to physical exercise, quality nutrition, glucose monitoring, medication, and social supports.

There have been very few studies on the psychosocial consequences of the COVID-19 pandemic on people with diabetes. In one study from Denmark, increased isolation negatively impacted participants’ quality of life and ability to carry out diabetes self-management practices [21, 22]. People with T2D in Denmark experienced increased anxiety, depression, and stress from COVID-19 [21, 22]. Our study found a similar phenomenon in Belize, suggesting that the ‘COVID-19 distress’ felt by people in the general population was compounded by the pre-existing mental chronicity burden understood as ‘diabetes distress’ for those with T2D, and this in turn was further intensified by the socio-economic context of living in the less developed, less resourced country of Belize. Previous literature has linked social and spiritual support to improved T2D self-management outcomes in Belize [2].

Danish research also pointed out the need for policymakers to differentiate between those who have their diabetes well-managed and those who do not, to create more nuanced policy around COVID-19 risk management, so as not to obstruct T2D self-management practices and subsequently, worsen T2D outcomes [21, 22]. Policymakers need to think carefully about how people can cope—or cannot cope well—with reduced appointments, access to medications, food markets, exercise venues, motivational and educational activities, religious and spiritual practices, and other self-management supports, especially those living with already limited resources, when for balanced risk management equations [2, 21, 22, 40].

Diabetes education strategies are clearly needed and wanted in Belize. Education is crucial for patients to gain the knowledge, skills, and abilities necessary to cope with their disease [34]. An American study on diabetes education showed education interventions to reduce incidences of T2D complications, hospital admissions, and readmissions, and to reduce lifetime costs while being well-accepted by patients [34]. An accessible T2D education program could eliminate the proportion of the growing prevalence of diabetes and its complications that is due to misunderstood pathogenesis and pathophysiology. As it stood, the lack of a comprehensive, nationwide education program fed into circulating misinformation, exacerbation of the prevalence of the disease and its complication, barriers to health-seeking behaviour, and barriers to screening and testing. Type 2 diabetes is a preventable disease with adequate education and supports.

Educational dissemination can occur over whatever media is accessible; a systematic review of T2D self-management education over cell phone messages demonstrated improved health outcomes [24]. A study in the United Kingdom found an online program called MyDesmond to be a useful tool to support people with type 2 diabetes with their self-management in the pandemic context [35]. A recent pilot of the program in Australia showed significant improvement of health outcomes through self-paced education via discussion forums, motivational sessions, goal setting and monitoring tools, incremental challenges, health trackers, an ‘Ask the Expert’ function, and a buddy system, all customized to promote behaviour change [35]. Another culturally-tailored intervention with African Americans living with T2D showed success with remote learning over Zoom during the pandemic [40]. These types of interventions could be adapted to the Belizean context; while not everyone can access a computer, many people have cell phones which can receive informational and motivational text messages. Investing in diabetes education is needed to curb rising prevalence and associated expenditures [3, 44].

Limitations of the study include lacking data from the Orange Walk district. The exact distribution of health care tiering across demographics is unclear, as is how this may have changed during the pandemic. Comparative analysis of T2D complications and interactions with COVID-19 before, during, and after the pandemic would be useful future research. We do not know how many people in which regions have access to phones, computers, or internet, but a survey to this end would be beneficial in discerning the most appropriate educational modalities. A strength of the study was its adaptability to the pandemic context, which also signals hope for interventions going forward. Strong international partnerships were key in succeeding in the research endeavours. Further research is needed on innovative educational interventions to curb the widespread societal costs of T2D in Belize and other low resource settings where people were hit the hardest by COVID-19.

## Conclusion

The qualitative data in this study was generated directly from people living with T2D in Belize. The findings outlined that people have been facing financial, informational, systemic, and structural barriers to optimal health and health care; these difficulties were exacerbated by the COVID-19 pandemic due to constraints restricting access to clinical care and self-management practices. To address these issues, there is need for: 1) extending public health care to all Belizeans; 2) a comprehensive, nationwide diabetes prevention and education program; and 3) a proliferation of innovative opportunities for physical, nutritional, economic, and psychosocial health and wellness. Recommendations for activities include sending informational and motivational text messages, creating a Belizean T2D cookbook and recipe calendar, televising educational workshops, making online tools more accessible, and mobilising support networks.

There are important implications for patients and providers, for policy and planning, and for research and development. More research is needed to support T2D education and self-management resources for those living with chronicity in Belize. Further assessment could determine more specific gaps in services, how to address the resultant inequities, and prevent expensive, life-altering complications. Research aimed at tackling the underlying issues and impacts of poverty and health inequity on T2D outcomes will be crucial future work.

## Data Availability

Data and material available conditionally upon request from corresponding author.
